# Management of Volkmann’s Ischemic Contracture: Case Series of 32 patients

**DOI:** 10.1051/sicotj/2021055

**Published:** 2021-11-11

**Authors:** Otman Benabdallah, Mohamed Shimi, Hicham Ait Benali, Ahmed Khamlichi, Rania Benabdallah

**Affiliations:** 1 Former Head of Department at El Kortobi and Mohamed V Hospital, Italian Hospital of Tangier Tangier Morocco; 2 Head of Department at Centre Hospitalier Universitaire of Tangier Morocco; 3 Mohamed V Hospital Tangier 90100 Morocco; 4 Centre Hospitalier Universitaire of Tangier Morocco; 5 Centre de Traumato-Orthopedie 3 rue Hopital Espagnol Tangier Morocco

**Keywords:** Compartment syndrome, Volkmann’s ischemic contracture, Muscle injury, Nerve injury

## Abstract

*Introduction*: Volkmann’s contracture condition is of high prevalence in our population and is linked to therapeutic faults. The treatment and its results are determined according to the severity of the lesions. *Methods*: This retrospective study was performed in three centers and was conducted over 30 years (1987–2018); it included 32 patients. The disabilities of the arm, shoulder and hand (DASH) score and the Weber test were used to evaluate the functional outcome looking at mid and long-term results. *Results*: Thirty-two patients were treated for Volkmann’s Ischemic Contracture (VIC). The age ranged from 4 to 58 years, with 19 patients aged under 15. Wrist fracture was the predominant cause in 16 cases. Fourteen patients obtained a completely functional hand, seven good functional results, four fair functional results, and seven poor results. *Discussion*: In comparison with other studies, we noticed significant differences: apart from the dominant male sex and right side, this is one large case series conducted over 30 years (1987–2018) looking at mid-and long-term results. All the patients presented with severe or moderate lesions on the first visit. In our study, the wrist fracture is predominant compared to elbow fractures and soft trauma. X-rays are especially helpful and are a first-line investigation for identifying displaced fractures and other associated lesions. Our study population is not large, and the treatment methods are varied, so it is impossible to provide statistically relevant correlations between the treatment method and outcome. But this work is based on the experience of more than 30 years, which makes it possible to help adequate decision making according to the state of the lesions. This study is a level IV case series.

## Introduction

**Figure 1:**
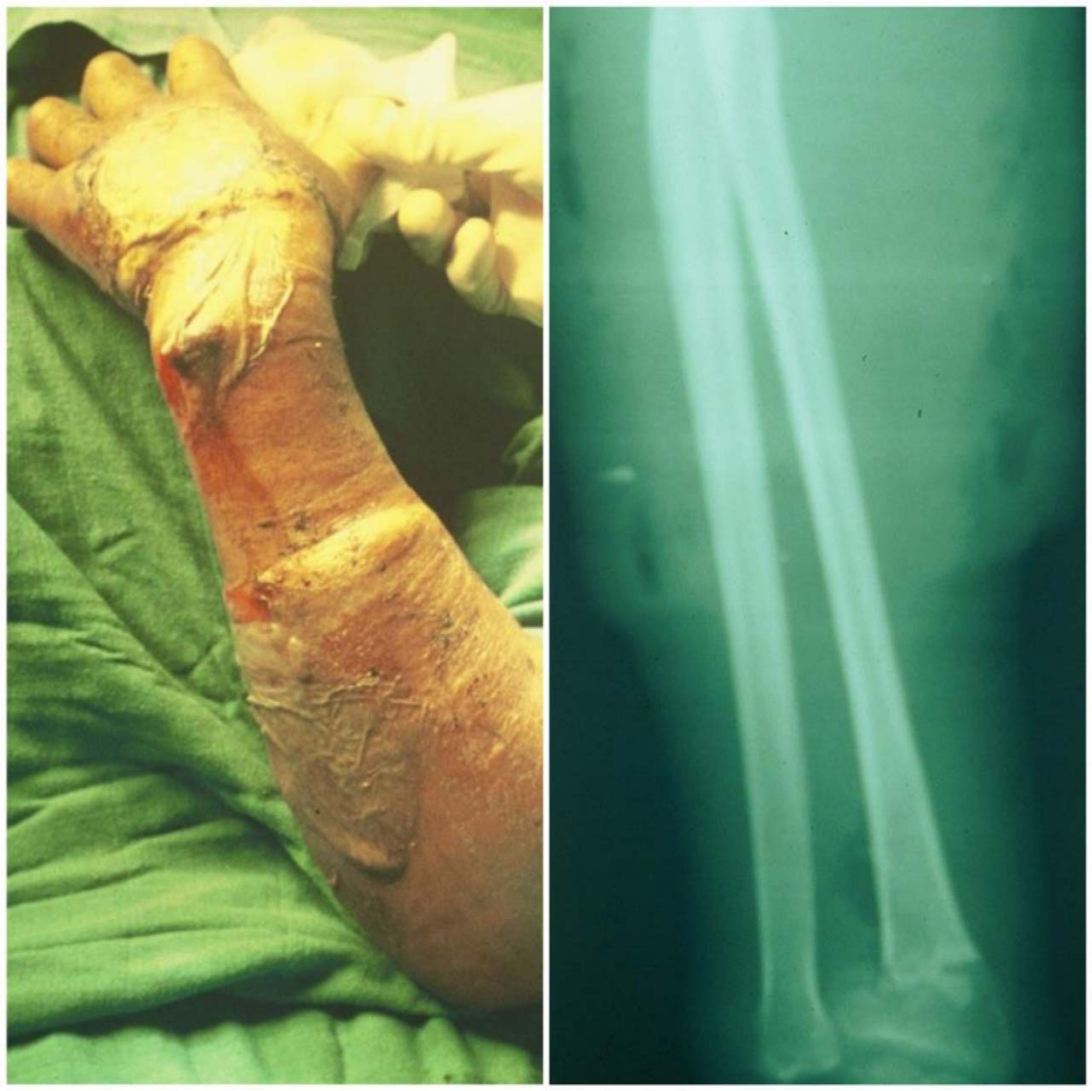
On the left figure it is about a 58-year-old woman with a severe case of Volkmann Ischemic Contracture complicated by an anaerobic bacteria infection; we can still see the traces of the bandage. The right picture is the same patient with an X-ray showing a wrist fracture and gas bubbles inside the soft tissue of the forearm, confirming gas gangrene.

**Figure 2:**
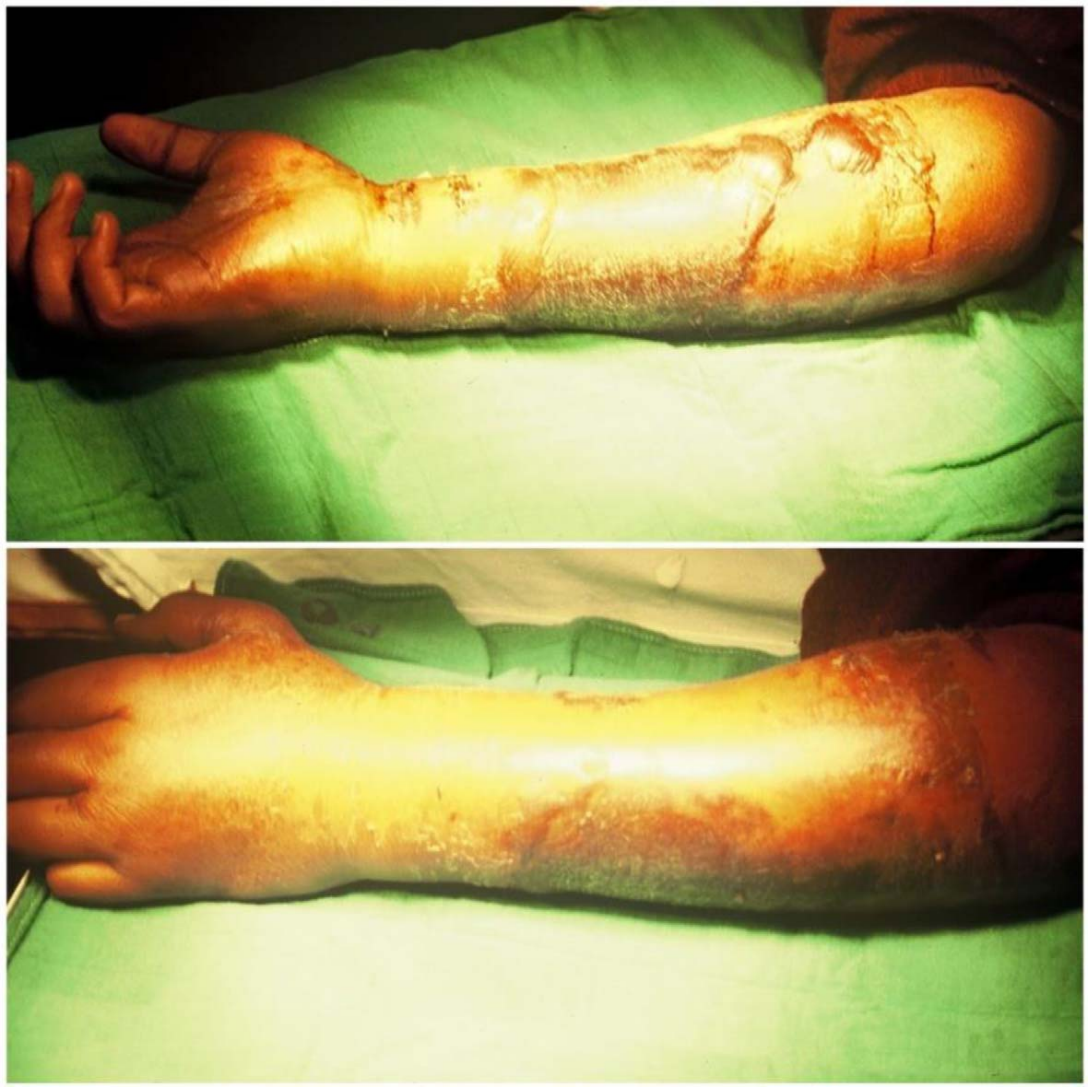
A 23-year-old girl presented a severe case of Volkmann Ischemic Contracture complicated by infection with common germs; the limb is swollen and has blisters. The cause is soft tissue trauma.

In 1881, Volkmann [1] described a condition of muscular ischemia, necrosis, and subsequent contracture of the forearm. Volkmann’s contracture occurs when swollen muscles in an inflexible envelope become ischemic when the capillary closing pressure is exceeded. The envelope in question consists of the fascia and bone surrounding the muscle, but skin and external restrictions such as encircling dressings and plaster splints may contribute to the situation [2–4]. It highlights that the muscle can only tolerate 4 h of ischemia before irreversible changes occur [2]. In our case, the bandage is a traditional material for immobilizing a fracture, composed of cloth, flour with water, and reeds; it is tied around the part of the member. The consequences for a patient are always disastrous. In an acute situation, severe pain results, but even if the patient complains excessively, the traditional healer is unaware of the danger of compartment syndrome or Volkmann’s contracture, which is why the urgently needed management is not provided, resulting in serious complications including anaerobic infection with gas gangrene (Figure 1), common bacterial infections, osteitis, and severe contractures characterized by necrosis of all flexor muscles and extensor muscles, which further complicates the treatment. In our series, Volkmann’s syndrome is observed in children, adolescents, and adults, resulting from fractures or soft tissue trauma (Figure 2). We must insist that a clear clinical presentation of Volkmann’s ischemic contracture (VIC) does not require further investigation to confirm the diagnosis, as this will only delay the urgent treatment needed. The choice of treatment depends on the course and severity of the contracture. For a mild case of VIC, an urgent fasciotomy is managed. In the long-term severe case, the surgical techniques [3, 4] consist of: excision of the necrotic muscle, lengthening of the tendon, sliding of the flexor muscle (Page-Scaglietti procedure), tendon transfer, carpal resection, shortening bone, and free muscle transplantation.

In sum, a better understanding of the pathogenesis of compartment syndrome has led to faster diagnostic and therapeutic measures and reduced the incidence of VIC [5–19].

We want to clarify that the main objective of this retrospective study was to assess the functional outcome in patients treated for VIC by looking at the mid-and long-term outcomes with different treatment methods.

Our study population is not large, and the treatment methods vary, so it is impossible to provide statistically relevant correlations between the treatment method and outcome. However, our experience and the results of our 30-year study can help decision-making in the treatment of patients with VIC.

## Method and material

**Figure 3:**
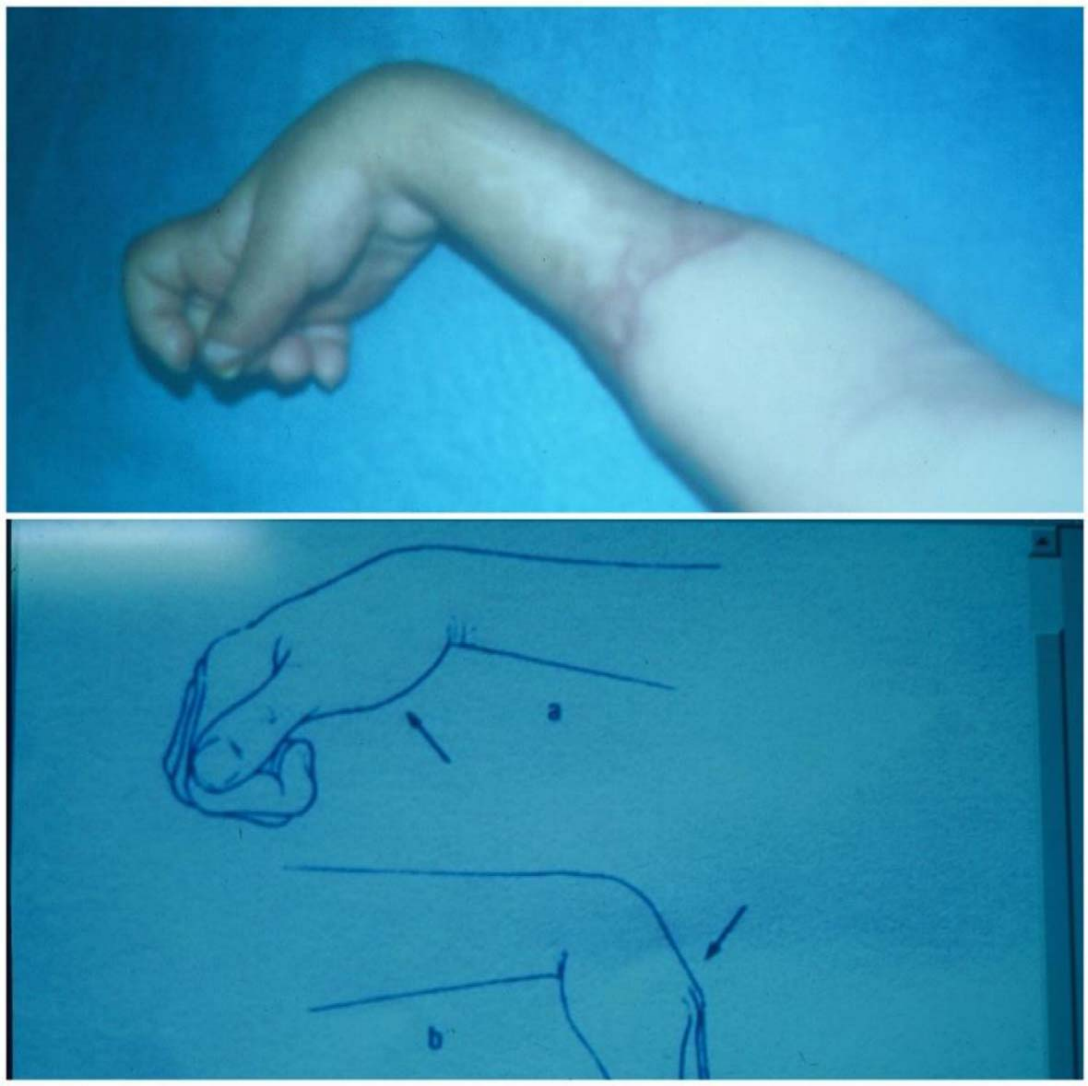
The passive stretch of the finger muscles. Increase in pain occurs when stretching the fingers.

**Figure 4:**
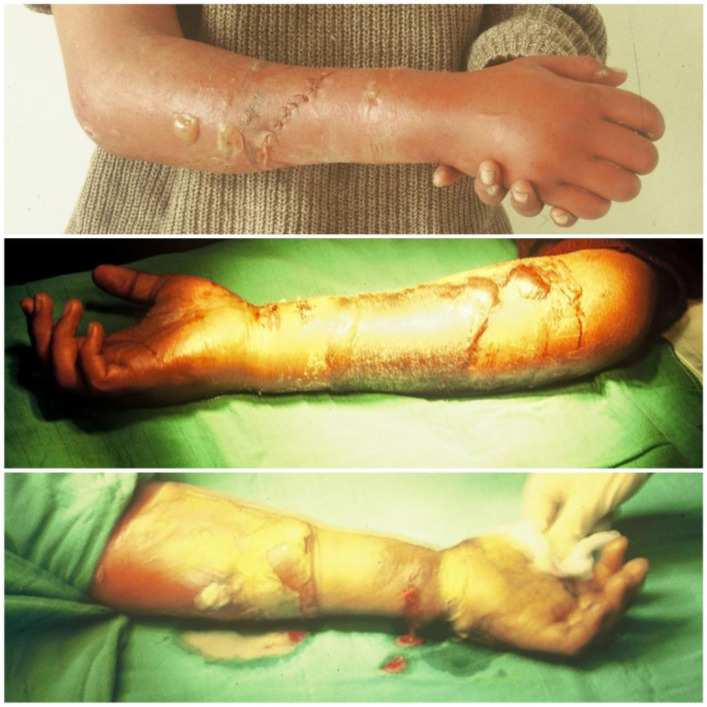
Three complicated cases: two Infections with common germs and one gangrene complication (bottom picture) at first assessment.

**Figure 5:**
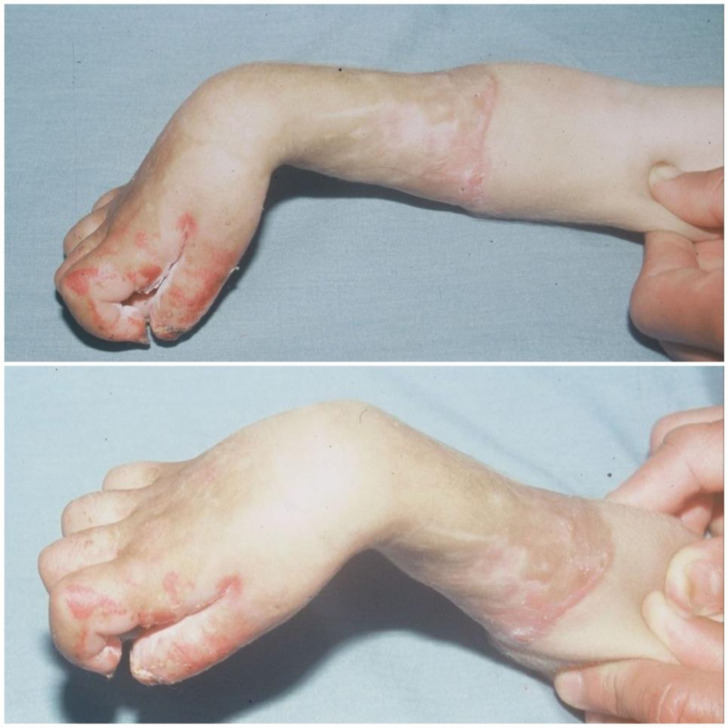
The specific sign, which is the indelible trace of the encircling material.

Among 37 patients, five files were unusable, therefore for the present study, 32 patients were selected to meet the following criterion: a clinical history of VIC established following inadequate treatment of compartment syndrome with an archaic encircling dressing manufactured by a traditional healer. This clinical history is characterized by a cardinal symptom which is severe pain out of proportion to the injury and features of ischemia such as pallor, paresthesia, loss of pulse, and paralysis. A useful clinical sign is increased pain during passive stretching of the finger muscles (Figure 3). Sometimes we see patients at the first assessment with infections or gangrene complications (Figure 4). In the long term, we observe contracture deformities of the fingers, hand, and wrist, and a specific sign that is the indelible trace of the encircling material (Figure 5). These lesions are classified into three levels of severity of Volkmann’s contracture: mild: contracture of the fingers with no or limited loss of sensitivity; moderate: all fingers are flexed, the wrist is stuck in flexion, and there is loss of sensitivity in hand and the presence of trophic lesions such as blisters and pressure sores; severe: all the muscles of the forearm that flex and extend the wrist and fingers are involved; there is a more or less important paralysis of the hand.

In our series, 17 of the patients were classified as moderate and 15 as severe.

Almost all of the patients were from disadvantaged social categories. However, there were two middle-class patients and, one young 14-year-old patient who was the son of a lawyer.

**Figure 6:**
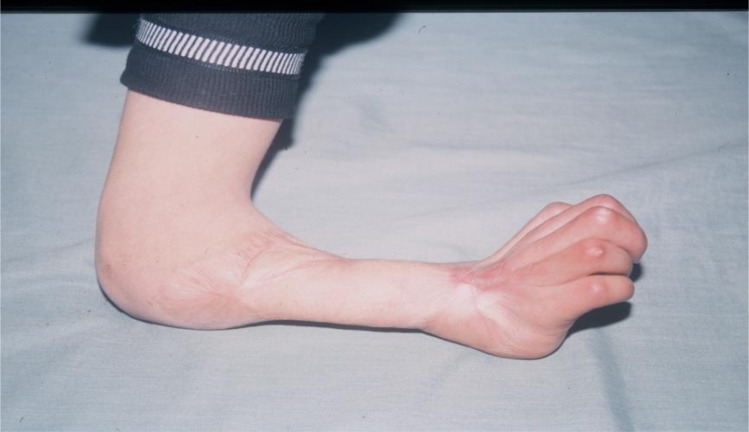
Severe case characterized by necrosis of flexor and extensor muscles.

The choice of treatment depends on the severity of the contracture. In moderate cases, the necrosis is confined to the flexor digitorum profundus, while in larger retractions, the contracture affects the flexor digitorum profundus, flexor digitorum longus, and sometimes flexor digitorum superficialis. On examination, a flexion deformity is noted in all the fingers, thumb, and wrist; there is sensory damage to the median and ulnar nerves. With neurological disorders, severe contractures are characterized by necrosis of all flexor muscles and sometimes extensor muscles (Figure 6). In mild and moderate levels, it will involve a fasciotomy. The muscle slide technique treats the severe forms; the muscle release depends on the extent of the muscle contracture. The release of the ulnar flexor is done on the outer edge of the anterior face of the ulna and the flexor digitorum profundus on their insertion in the interosseous membrane. In the most severe cases, where the flexor digitorum longus of the thumb is affected, the release is more extensive and includes an avulsion of the pronator teres of the radius. The flexor digitorum longus is freed from the radius by subperiosteal disinsertion. This allows an additional 3–4 cm of slippage. Neurolysis is performed in accordance with the lesions found. In some cases, the muscle slide technique is ineffective. It may prove necessary to perform excision of necrotic muscles (although we keep these excisions to an absolute minimum) with neurolysis and tendon transfer. If no muscle is available, we will carry out a free muscle graft. The onset of muscle contraction is noted in the third month, and significant flexion of the fingers is possible after five or six months.

Sixteen fasciotomy procedures were performed in patients with moderate contracture, but generally, they were associated with neurolysis, tenolysis, vascular liberation, and sometimes muscle excision. The interval between the establishment of the contracture and surgical management was between one and three weeks. Although fingers and wrist mobility and sensitivity decreased after establishing the contracture, all patients improved rapidly in function over the first three to six weeks.

Nine muscle slide operations were performed in patients with severe ischemic contracture; this procedure modifies the active mobility sector of the retracted muscles in the fingers to improve hand function.

Two digital amputations were carried out in patients with digital necrosis; the results were poor.

Five combinations of operative techniques were used in patients with severe ischemic contracture, with disappointing results. A two-year-old child underwent two fingers amputation (elsewhere), a slide operation at age eight, and a wrist arthrodesis at age 18; the result was very poor. A 24-year-old patient had a combination of fasciotomy, tenolysis and neurolysis, tendon transfer, and later bone curettage for osteomyelitis; motor function did not improve, and the result was poor. One patient underwent slide surgery, a carpal resection with minimal results, and then a wrist osteotomy to improve function. A ten-year-old patient underwent slide surgery; he developed cutaneous retraction in the elbow and wrist as he grew, which needed a Z-Plasty. Transplantation was performed in a patient with severe contracture and significantly necrotic and fibrotic muscles, for which a slide operation was not effective. First, necrotic muscular excision with neurolysis was required, followed by the transplantation; the prognosis was good.

The disabilities of the arm, shoulder and hand (DASH) score and the Weber test [13] were used to evaluate the functional result.

The result was rated as a percentage of performance relative to the uninjured hand and was rated as excellent, good, fair, and poor (function reduced by 1/4, 1/2, 3/4. or no function at all).

The sensitivity coefficient of each finger was 0.75, 0.5, 0.25, and 0 for a total absence of sensitivity in the finger. It was classified into two groups: Excellent–Good (E–G) and Fair–Poor (F–P).

## Results

Thirty-two patients were treated between January 1987 and December 2018 for VIC (see data in Table 1). The age ranged from 4 to 58 years, with 19 patients under 15 years. There were 20 male patients and 12 females. Twenty-three patients were affected on the right hand and 9 on the left hand. The cause of the condition was a wrist fracture in 16 cases, an elbow fracture in eleven cases, and soft limb trauma in five cases.

**Table 1:** Characteristics of patients, options, and results of treatment.

	Case	Age–Sex	Cause	Side	Level	Duration	Findings	Treatment	Follow up	Result	Sensitivity	Score (%)
Fasciotomy	1	57 F	Wrist	L	Severe	3 weeks	Gangrena	Fasciotomy	20 years	Excellent	E–G	13.60
	2	15 M	Wrist	R	Moderate	2 weeks	Infection	Fasciotomy	2 years	Excellent	E–G	0.00
	3	15 M	Elbow	L	Moderate	2 weeks	Infection	Fasciotomy	4 years	Excellent	E–G	0.00
	4	19 F	Wrist	R	Moderate	3 weeks		Fasciotomy	2 years	Excellent	E–G	0.00
	5	23 F	Wrist	R	Moderate	2 weeks		Fasciotomy	3 years	Excellent	E–G	0.00
	6	14 M	Elbow	R	Moderate	2 weeks		Fasciotomy	4 years	Excellent	E–G	0.00
	7	13 M	Wrist	L	Moderate	3 weeks		Fasciotomy	2 years	Excellent	E–G	0.00
	8	22 F	Elbow	R	Moderate	2 weeks		Fasciotomy	2 years	Excellent	E–G	0.00
	9	28 M	Wrist	R	Severe	3 weeks		Fasciotomy	3 years	Good	E–G	31.80
	10	4 F	Wrist	L	Moderate	2 weeks		Fasciotomy	2 years	Excellent	E–G	9.10
	11	13 M	Elbow	R	Moderate	2 weeks		Fasciotomy	4 years	Good	E–G	25.00
	12	16 M	Soft	R	Moderate	3weeks		Fasciotomy	2years	Excellent	E–G	22.70
	13	13 M	Wrist	R	Moderate	4 weeks		Fasciotomy	3 years	Good	E–G	29.50
	14	22 M	Wrist	L	Moderate	2 weeks		Fasciotomy	2 years	Excellent	E–G	18.20
	15	6 F	Soft	R	Severe	5 weeks		Fasciotomy	4 years	Good	E–G	47.70
	16	9 M	Elbow	R	Severe	4 weeks		Fasciotomy	3 years	Good	E–G	34.10
Slide procedure	17	23 M	Elbow	R	Severe	5 weeks		Slide procedure	9 years	Fair	E–G	63.60
	18	26 F	Wrist	R	Severe	3 weeks		Slide procedure	2 years	Good	E–G	40.90
	19	9 M	Elbow	R	Moderate	3 weeks		Slide procedure	3 years	Good	E–G	38.60
	20	7 F	Wrist	R	Moderate	4 weeks		Slide procedure	3 years	Good	E–G	40.20
	21	11 M	Soft	L	Severe	4 weeks		Slide procedure	5 years	Fair	E–G	54.50
	22	8 F	Wrist	L	Severe	3 weeks		Slide procedure	3 years	Good	E–G	47.70
	23	11 M	Elbow	R	Severe	3 weeks		Slide procedure	2 years	Fair	E–G	52.30
	24	14 M	Soft	R	Severe	2 weeks		Slide procedure	2years	Fair	E–G	56.80
	25	9 M	Wrist	L	Moderate	3 weeks		Slide procedure	3 years	Fair	E–G	54.50
Combined procedure	26	27 M	Soft	R	Severe	8 weeks		Fasciotomy-carpus	4 years	Good	E–G	36.40
								Resection-muscle				
								Transplantation-				
								Wrist osteotomy				
	27	4 F	Elbow	R	Severe	5 weeks		Digital amputation	25 years	Poor	F–B	77.30
								Fasciotomy				
								Wrist arthrodesis				
	28	10 M	Elbow	L	Severe	7 weeks		Slide procedure	16 years	Poor	E–G	65.90
								Z plasty				
								Tendon transfer				
	29	24 M	Elbow	R	Severe	5 weeks	Osteitis	Slide procedure	9 years	Poor	F–B	77.30
								Bone curettage				
	30	28 M	Wrist	R	Severe	4 weeks	Infection	Slide procedure	3 years	Fair	E–G	52.30
								Carpal resection				
								Wrist osteotomy				
Digital amputation	31	5 F	Wrist	R	Severe	4 weeks		Digital amputation	2 years	Poor	E–G	77.30
								Tendon transfer				
	32	27 F	Wrist	L	Severe	5 weeks		Digital amputation	2 years	Fair	E–G	72.70

The patients were evaluated with a mean interval range between treatment and assessment of 2–25 years (median 8 years).

Overall, 14 patients obtained a completely functional hand, seven good functional results, four fair functional results, and seven poor results.

The rating scores for our management and treatment results are summarized in Table 1. In the fasciotomy category, which included 16 patients (12 severe and 4 moderate levels), 11 patients were rated excellent and 5 good. The overall score was, on average, 14.48%. Of nine patients treated with the slide procedure (Page-Scaglietti) for six severe and three moderate cases, four were rated good and five fair. The overall score was, on average, 49.90%. In the combined procedure, which included five severe cases, one was rated as good, one as fair, and three as poor. The overall score was 61.84%. In the amputation category for two severe cases, one patient showed a fair result, and another poor, with an overall score of 75%.

For the four categories, the overall score was, on average, 50.30%.

Almost all of the patients improved sensitivity except in two cases, where the sensitivity was rather poor.

Comparison of our results showed a better score for the fasciotomy followed by the slide procedure than the combined treatment.

## Discussion

Volkmann’s ischemic contracture (VIC) is not uncommon, reflecting a high prevalence of this disease in our population. In literature, a search in PubMed found 1107 articles published since 1903, eight of which were last year, 2020.

The study included 32 patients, ranging in age from 4 to 58 years old, patients relatively young, of which 19 patients were less than 15 years old.

Patients were followed clinically for periods ranging from a minimum of 2 years to a maximum of 25 years (median 8 years).

In comparison with other studies [6, 17–19], we noticed significant differences. First, it is a large case series, conducted over 30 years (1987–2018), and looking at the mid-and long-term results. Apart from the predominance of the male sex and the right side and as other authors have also pointed out [17–19] on this subject, the more frequent involvement of men than women can be attributed to the greater tendency of men to participate in risky activities which predispose them to initial traumatic events. The cause of this condition is also different in our study, with a predominance of wrist fractures (16 cases, compared to 11 cases of elbow fracture and five soft limb injuries). In literature [6, 13, 14, 19], VIC secondary to supracondylar fractures is found in 10.07% to 60% of cases, while it is secondary to fractures of both of the forearm bones in 54.6% of cases. Another difference in our study is the presence of gas gangrene in a 57-year-old woman and two infections with common germs, and a case of osteitis in a 24-year-old man. These cases are unusual, and we have not found similar cases in the literature. Another difference is about X-rays useful for identifying displaced fractures and associated lesions (such as bubbles inside soft tissue, confirming gas gangrene as in Figure 1) or confirming osteitis secondary to infected Volkmann Ischemic Contracture. Imaging studies such as magnetic resonance imaging (MRI) and computed tomography (CT) may prove to be supportive in the diagnosis of VIC [19], but they are not considered as first-line investigations.

Regarding the treatment and unlike some authors who privilege a type of treatment, Page Scaglietti for Griffart et al. [19] or neurolysis for Meena et al. [18], free muscle transfer or neurolysis-tenolysis for Ultee, our treatment is on request depending on the severity of the lesions. The primary emergency for compartment syndrome leading to VIC is fasciotomy (even for severe cases) performed via the anterior approach as recommended by Roy Camille [3]. In this study category, which included 12 moderate and 4 severe cases, the assessment score ranged from 0.00% to 47.70%, with an average of 14.48%. The mean age was 18 years (with a range of 4–58 years) with nine patients under 15 years old. The duration of disability was, on average, 2.7 weeks. The outstanding result in this category is a 57-year-old woman with VIC complicated by gas gangrene infection. The result of treatment was excellent (13.60%) despite the severity of the lesions. Our study should emphasize that fasciotomy is effective for ischemic Volkmann contractures complicated by anaerobic or banal germ infections. The outcome of fasciotomy for compartment syndrome leading to Volkmann’s contracture depends on the duration and intensity of early VIC. Rapid fasciotomy usually leads to minimal sequelae (16 excellent and good results in our series).

Regarding the slide procedure, the duration of disability was on average 3.3 weeks; nine patients with an average age of 11 years obtained four and five good results (mean score 49.90%). In this segment, seven were children under the age of 15 who had undergone slide surgery due to the severity and duration of the disability. The muscle sliding technique is satisfactory when the muscles are retracted but active; it modifies the sectorial mobility of the fingers to obtain a good functioning of the hand.

Otherwise, five patients underwent alternative combined procedures for their treatment, depending on the complications that arose: fasciotomy, muscle transplantation, carpal resection, slip procedure, wrist osteotomy, wrist arthrodesis, and Z-Plasty. Overall scores for these five patients averaged 61.84%, with one good, one fair, and three bad.

It may also be necessary to perform nerve grafting, reinnervation, and/or tendon grafting to reactivate paralyzed tendons. In many cases, wrist extensor muscles and tendons are not available as tendons transfers due to their involvement in the initial process, so patients with severe contractures require complex reconstruction using free muscle transfer.

In total, we obtained 21 satisfactory results and 11 unsatisfactory results.

For the four categories, the overall score was, on average, 50.30%.

Almost all of the patients improved sensitivity except in two cases, where the sensitivity was relatively poor.

In comparison with other studies, in 2017, Griffart et al. [19] reported seven cases treated with the Page-Scaglietti procedure, all of which had satisfactory results, even with a long delay after injury (63.6 months). In 2005, Ultee et al. [6] reported 12 good and reasonable results and 12 unsatisfactory and poor cases; the best results were attributed to free transfer of innervated microvascular muscles in seven patients and to neurolysis-tenolysis or tendon transfer in five patients. The delay after injury was, on average, 32.84 months. In 2016, Meena et al. [18] reported on a study in which the treatment was neurolysis of the median and ulnar nerves, and the results were divided into two groups. One group consisted of cases operated on within six months of the onset of VIC. The second group consisted of cases operated on more than six months after the onset of Volkmann’s contracture. The results revealed no statistically significant difference between the two groups, although both had significant improvement in motor and sensory recovery. In 2020, Saaiq [17] reported a series of 37 patients. The most common underlying cause was the treatment of forearm fractures with traditional bonesetters. Most of the patients were treated with a combination of procedures, including tendon transfers, excision of fibrotic muscles, tenolysis, neurolysis of the median and ulnar nerves. Tendon transfers were the most common corrective procedure.

**Figure 7:**
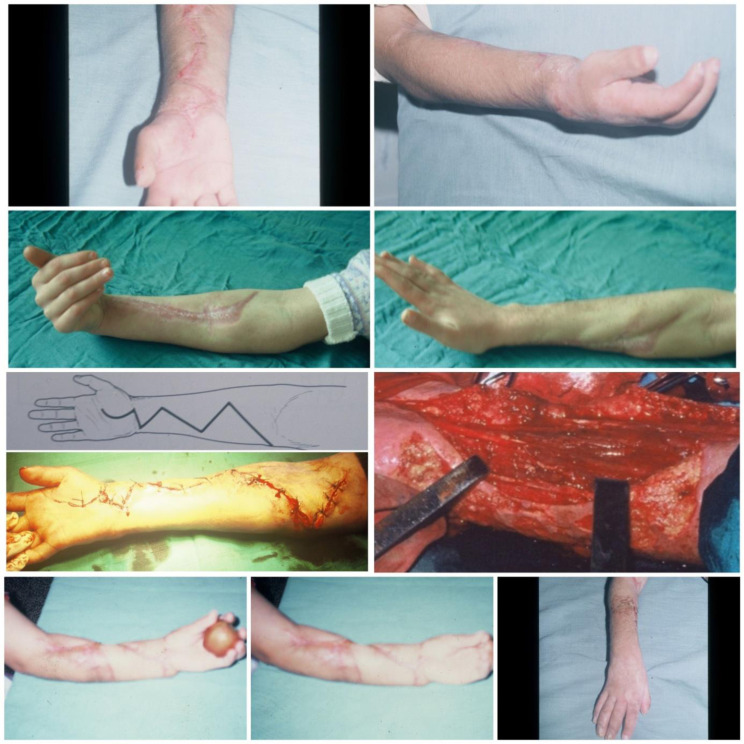
Five severe cases operated by fasciotomy, neurolysis, and tenolysis, which results are good or excellent.

**Figure 8:**
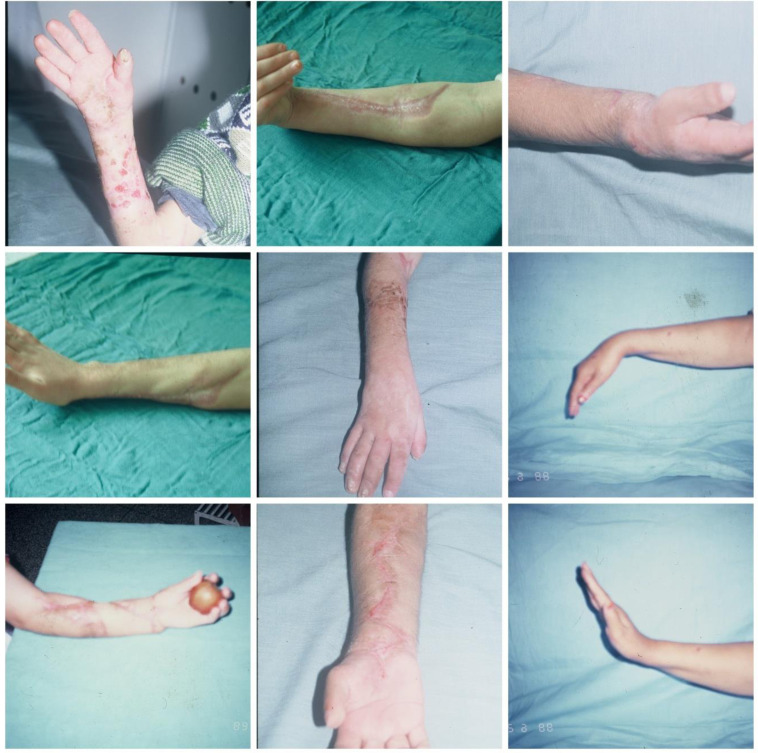
Results of various cases operated with different surgical procedures:slide procedure, fasciotomy, osteotomy, transplantation; the goal of surgery is to maximize limb function.

The timing of intervention for established contracture is a subject of debate. Seddon [13] recommends that it be carried out after three months, while Tsuge [14] recommends waiting six months. Meena et al. [18] reports that the timing of the surgery did not play a role in the outcome. In our experience, surgery on retractions is done on request, and an early response is advisable to avoid thinning of the nerves and developing joint stiffness (Figures 7 and 8).

Moreover, alternative treatment by traditional healers [17, 20–22] still exists, which suggests an underestimation of the devastating effects of ischemia if it is not recognized and treated in time. The non-recognition of Volkmann’s contracture is the main factor preventing the urgent management it requires. Unfortunately, there is no litigation against healers in our country and probably in other developing countries.

We must always insist on prevention, which is the best treatment. This highlights the importance of keeping a watchful eye for signs and symptoms of compartment syndrome and VIC in patients with limb fractures and soft trauma treated with bandages and tight dressings.

In summary, most authors [6, 17–19] report improved results when performing an open repair, whatever the procedure adopted.

About these data, we notice: that VIC is still a hot topic; its management is complex; the operative indications are to tackle strategic lesions as soon as possible; this work is based on an experience of more than 30 years which makes it possible to help to take adequate decisions according to the state of the lesions.

There are limits to this study: we did not report all possible types of cases, but our study included unusual cases not described in the literature; there are no ready-made solutions applicable in all circumstances; Comparison with other studies was sometimes difficult given their different design; Our study population is not large, and the treatment methods are varied, so it is not possible to provide statistically relevant correlations between the treatment method and the result.

To conclude, Volkmann’s syndrome is a problematic condition whose treatment is determined according to the severity of the lesions. The results depend on the reversibility of the lesions, and the prognosis is uncertain.

## Conflict of interest

The authors do not have any conflict of interest.

## Funding

This research did not receive any specific funding.

## Ethical Approval

Ethical approval was not required.

## Informed consent

This article does not contain any studies involving human subjects.

## Authors contributions

Otman Benabdallah: Conceptualization, Investigation, Writing Original Draft; Mohamed Shimi: Formal Analysis, Investigation; Hicham Aitbenali: Data Curation, Investigation; Ahmed Khamlichi: Investigation; Rania Benabdallah: Visualization.
